# The role of intestinal microbiota and microRNAs in the anti-inflammatory effects of cranberry: from pre-clinical to clinical studies

**DOI:** 10.3389/fnut.2023.1092342

**Published:** 2023-05-23

**Authors:** Amel Taibi, Zoe Lofft, Bianca Laytouni-Imbriaco, Elena Maria Comelli

**Affiliations:** ^1^Department of Nutritional Sciences, University of Toronto, Toronto, ON, Canada; ^2^Joannah and Brian Lawson Centre for Child Nutrition, University of Toronto, Toronto, ON, Canada

**Keywords:** cranberry, polyphenols, proanthocyanidin, intestinal microbiota, intestinal health, inflammation, microRNA

## Abstract

Cranberries have known anti-inflammatory properties, which extend their benefits in the context of several chronic diseases. These benefits highly rely on the polyphenol profile of cranberries, one of few foods rich in A-type proanthocyanidin (PAC). A-type PAC comprises flavan-3-ol subunits with an additional interflavan ether bond in the conformational structure of the molecule, separating them from the more commonly found B-type PAC. PACs with a degree of polymerization higher than three are known to reach the colon intact, where they can be catabolyzed by the gut microbiota and biotransformed into lower molecular weight organic acids that are available for host absorption. Gut microbiota-derived metabolites have garnered much attention in the past decade as mediators of the health effects of parent compounds. Though, the mechanisms underlying this phenomenon remain underexplored. In this review, we highlight emerging evidence that postulates that polyphenols, including ones derived from cranberries, and their metabolites could exert anti-inflammatory effects by modulating host microRNAs. Our review first describes the chemical structure of cranberry PACs and a pathway for how they are biotransformed by the gut microbiota. We then provide a brief overview of the benefits of microbial metabolites of cranberry in the intestinal tract, at homeostasis and in inflammatory conditions. Finally, we discuss the role of microRNAs in intestinal health and in response to cranberry PAC and how they could be used as targets for the maintenance of intestinal homeostasis. Most of this research is pre-clinical and we recognize that conducting clinical trials in this context has been hampered by the lack of reliable biomarkers. Our review discusses the use of miRNA as biomarkers in this context.

## 1. Introduction

The American Cranberry (*Vaccinium macrocarpon*) is the most consumed species of cranberry among the four known types in North America [reviewed in Feghali et al. (1)]. For decades, cranberries have attracted attention in the field of nutritional sciences because of their abundant, unique polyphenol profile that renders them as powerful antioxidants and modulators of inflammation (2). Polyphenols are also increasingly regarded as potential prebiotic compounds. This is because polyphenols of higher molecular weight that are too large to be absorbed in the small intestine, are then directed to the colon, where they are catabolized into organic acids by the gut microbiota; these become available for host absorption and thus, could mediate the health effects of polyphenols (3). Cranberry and its polyphenols also influence the composition of the gut microbiota, thereby potentially affecting its function and production of secondary metabolites from various dietary substrates. Both cranberry components and the gut microbiota affect the intestinal gene expression program. In particular, evidence has emerged that these effects manifest transcriptionally as well as post-transcriptionally via microRNAs (miRNAs) (4–6). It is thus possible that cranberry and the microbiota interact to affect host miRNAs and that this underlies downstream effects. The aim of this review is to provide a comprehensive assessment of the reciprocal interaction between dietary cranberry and the gut microbiota and to discuss the role of intestinal miRNAs in this context.

## 2. Polyphenols in cranberry

### 2.1. Chemical structure

**Figure 1:**
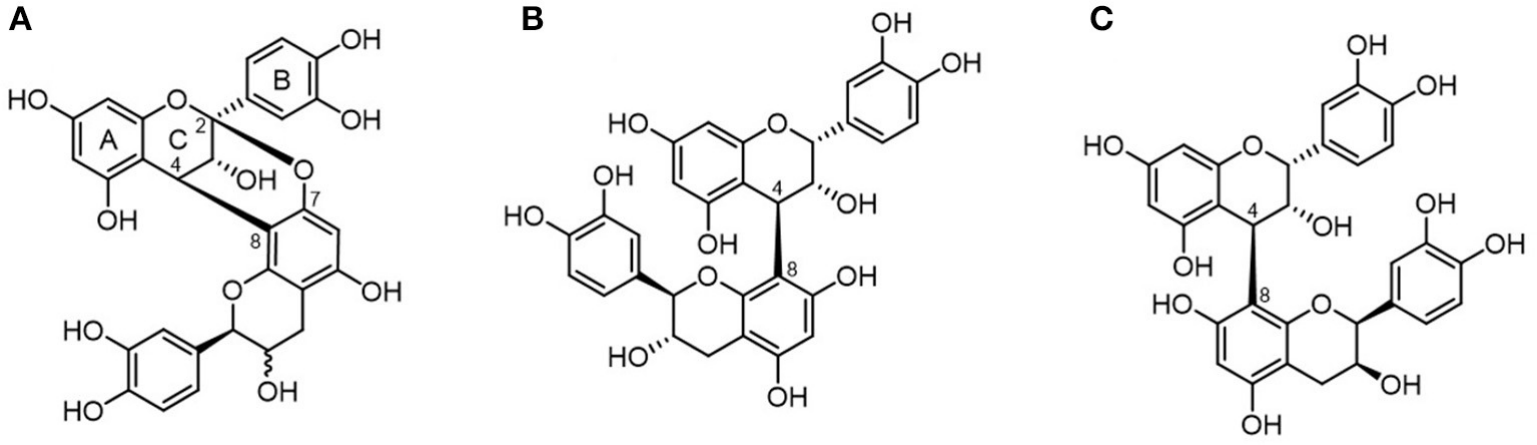
Chemical structure of proanthocyanidins. PACs are differentiated into A-type **(A)** proanthocyanidin A and B-type **(B)** proanthocyanidin B1 or **(C)** proanthocyanidin B2 depending on their interflavanic linkages.

In cranberry juice, the four main phenolic classes identified include phenolic acids and three flavonoid classes: anthocyanins, flavonols, and flavan-3-ols (7). About 85% (by weight) of the total flavan-3-ols are represented by proanthocyanidins (PACs). Cranberry PACs are typically formed by epicatechin subunits (flavan-3-ol monomers) with an average degree of polymerization (DP) of four to seven, which can extend up to 12 (8, 9). Opposite to most fruits PACs which contain B-type bonds (typically linked through C4 → C8 or C4 → C6, cranberries are among the richest sources of A-type PACs (10), with most PACs containing at least one A-type linkage (11). A-type bonds are doubly linked, containing an additional ether bond between the C2 of the upper unit of one heterocyclic ring, to the oxygen-bearing C5 or C7 on the lower unit awarding the molecule a degree of conformational inflexibility (Figure 1).

### 2.2. Consumption, bioavailability and microbial metabolism

#### 2.2.1. Consumption

Representative surveys of the European and American populations have estimated PAC daily intake to range between 95 to 160 mg/day, with marked differences among countries and dietary habits (12, 13). A 240 ml serving of a 27% juice cranberry cocktail contains an average of 38 mg of PAC [reviewed in Blumberg et al. (14)]; this amount corresponds approximately to the recommended daily intake of 36 mg/day that was shown to prevent urinary tract infections [reviewed in Howell et al. (15)]. Clinical studies have altered the composition of cranberry juice to contain more PAC, ranging from 77 to 118 mg per 240 ml serving in doubly concentrated 54% cranberry juice (16, 17), up to 922.2 mg per 240 ml serving in 117% cranberry juice (18). So theoretically, based on a colonic volume estimate of 561 ml (19), the consumption of a serving of PAC-enriched cranberry beverage could result in colonic exposure to PAC in concentrations ranging from 137 μg/ml to as high as 1,644 μg/ml. Colonic PAC is available for microbial metabolism (20) which could ultimately affect the magnitude of exposure across individuals.

#### 2.2.2. Bioavailability

Several studies have reported that cranberry polyphenols and their metabolites are bioavailable to the host. A variety of phenolic acids, flavonoids and their metabolites have been detected in human plasma and urine following acute or prolonged consumption of cranberry juice (17, 21–23). Hydrosoluble PACs are the only PAC molecules available for absorption and become accessible to the enterocyte surface in the small intestine. The absorption of non-soluble PACs depends on their DP, MW, and structure. An *in vitro* study showed that A-type PACs with lower DP (dimer, trimer and tetramer) can transverse the intestinal monolayer at low rates (24). Intestinal epithelial cells are important sites of PACs modification; these polymers undergo methylation and glucuronidation processes, producing methylated and glycosylated PACs with higher bioavailability (25). Polymers reach the colon intact where they are metabolized by the gut microbiota in a diverse range of metabolites (25, 26), with different hydroxylation profiles and aliphatic side chain lengths, such as phenylacetic, phenylpropionic, and phenylvaleric acids (3, 27, 28), which are available for absorption by colonocytes.

### 2.3. PACs metabolism by the microbiota

**Figure 2:**
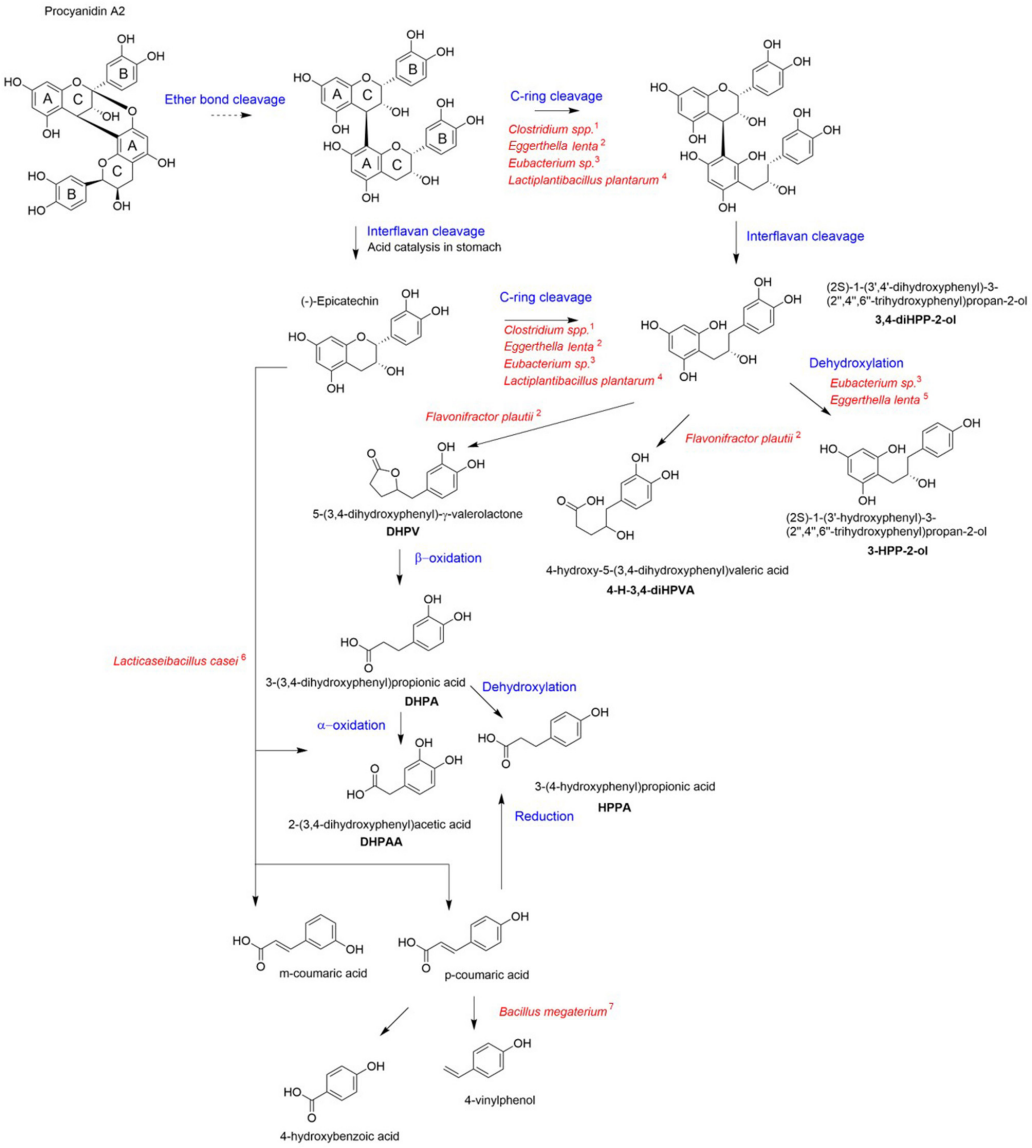
Proposed pathways for cranberry PAC microbial catabolism. Blue indicates the chemical reaction for each step and red corresponds to the bacterial taxa involved in the metabolite formation for each step. The catabolic pathway was generated via integration of published data [1: Corrêa et al. (36); 2: Kutschera et al. (37); 3: Wang et al. (38); 4: Sánchez-Patán et al. (39); 5: Jin and Hattori (40); 6: Li et al. (41); 7: Torres and Rosazza (42)].

The gut microbiota is an indispensable player for the processing of ingested food. The functional potential of the microbiota is estimated to be encoded in millions of genes (29), many of which are necessary to metabolize otherwise undigestible dietary components that reach the colon. Specifically, in the colon, the backbones of non-absorbed PACs undergo a series of bacterial enzymatic transformations resulting in the generation of less complex compounds. Figure 2 presents a compendium of these reactions that include C-ring cleavage, dihydroxylation and decarboxylation. Once absorbed, the microbial metabolites of PACs are transported through the portal vein to the liver, where they undergo further metabolism by phase II enzymes (30) before being released into systemic circulation. There is currently limited research to identify gut microbial taxa involved in the catabolism of cranberry polyphenols. Different bacterial genera have been reported to initiate the metabolism of flavan-3-ols, including *Eubacterium, Flavonifractor, Eggerthella, Lactobacillus*, and *Enterococcus* (20, 31, 32). *Clostridium coccoides, Eubacterium* spp*., Eggerthella lenta, Adlercreutzia equolifaciens, Slackia equolifaciens*, and lactobacilli species carry PAZymes (polyphenol-associated enzymes) (33). PACs first undergo partial acid-catalyzed interflavan cleavage to form procyanidin dimers epicatechins units, which are then transformed by colonic bacteria. *Eggerthella lenta, Clostridium spp*. and *Eubacterium sp*. are involved in opening the C-rings in epicatechins (34); following C-ring cleavage, *Flavonifractor plautii* further converts this intermediate into 5-(3′-4′-dihydroxyphenyl)-γ-valerolactone through A-ring breakdown and lactone formation (34, 35) (Figure 2).

*In vitro* microbiota fermentation of cranberry PACs was shown to produce higher amounts of 2-(3′-hydroxyphenyl)-acetic acid compared to apple (43). In comparison to grape seed polyphenols, cranberry PACs were catabolized to a greater extent and produced distinctly high amounts of 3-(3′,4′-dihydroxyphenyl)-propionic acid, 3-(4-hydroxyphenyl)-propionic acid (HPPA); 3,4-dihydroxyphenylacetic acid (DHPAA), and phenylpropionic acid (20). Both HPPA and DHPAA metabolites were reported to be produced from A-type PAC *in vitro*, using a pig cecum model (44). Moreover, HPPA was identified as the most highly produced metabolite from the bioconversion of PAC using *Lacticaseibacillus rhamnosus* (32). Inter-individual variability exists in the production of metabolites from cranberry polyphenols (23, 45), which may relate to bacterial PAZymes polymorphism, (46), host gut microbiota composition and diet (47). Nonetheless, PAC metabolites have been consistently detected in high amounts in human plasma or urine after consumption of cranberry juice. HPPA was identified as the most abundant polyphenol metabolite in plasma of healthy young individuals (23). Similarly, DHPAA has been found in high amounts in the urine of healthy older adults after acute cranberry juice exposure (22), as well as in overweighted adults who received a low-calorie cranberry extract beverage for 8 weeks (17).

Interestingly, the ingestion of cranberry modifies the composition of the microbiota, as shown in pre-clinical and clinical studies including both health and disease conditions (Table 1). The limited number of studies makes it difficult to identify a common pattern of microbial responses. Though, overall, the effect appears to favor a health-compatible microbial phenotype. This is sometimes accompanied by a measured physiological benefit, such as in a mouse model of colitis where cranberry powder administration reversed the microbiota changes found in inflamed mice and resulted in decreased colitis severity and production of pro-inflammatory cytokines (48). Taken together, these findings suggest that the bi-directional interaction between cranberry and the gut microbiota is an important determinant of cranberry benefits.

**Table 1:** Summary of relevant studies reporting gut microbial taxa that respond to cranberry.

**Type of study**	**Experimental model**	**Treatment**	**Main microbiota findings**	**Reference**
Pre-clinical	Eight-weeks old C57BL/6J male mice (*n* = 12/group)	C57BL/6J mice were fed either a chow or a High Fat-High Sucrose (HFHS) diet. HFHS-fed mice were gavaged daily either with vehicle (water) or cranberry extract (CE, 200 mg/kg) for 8 weeks	-CE+HFHS: ↑*Akkermansia muciniphila*,	Anhê et al. (49)
	Eight-weeks old C57BL/6J male mice (*n* = 8–10/group)	Mice received either a Chow or a HFHS diet for 13 weeks to induce obesity Mice were then treated either with Cranberry Extract (CE, 200 mg/kg), Chow+CE, HFHS+CE or vehicle (Chow, HFHS) for 8 weeks	-CE+HFHS ↓ Firmicutes/Bacteroidetes ratio and ↑*Akkermansia muciniphila, Coprobacillus*, and *Barnesiella*	Anhê et al. (50)
	Six-weeks-old CD-1 mice (*n* = 10/group)	Mice received either control AIN-93G diet or 1.5 % (w/w) freeze-dried whole cranberry powder (CP) mixed with AIN-93G diet for 12 weeks The dietary dose of cranberry was equivalent to an oral dose of 7.5 g of whole CP or 65 g fresh cranberry fruits per day for humans Colitis induced by dextran sodium sulfate (DSS)	-Reversed the change of gut microbiota in colitis mice (↑*Lactobacillus* and *Bifidobacterium*; ↓ harmful bacteria, i.e *Sutterella* and *Bilophila)*	Cai et al. (48)
	Eight-weeks old C57BL/6 male mice (*n* = 6)	Mice were inoculated with human microbiota (25 human-origin commensal species-GnotoComplex 2.0 flora) After 14 days, mice received either with Cranberry Juice Extract (CJE, 200 mg/kg) for 10 days	-CJE: ↑*A. muciniphila and Clostridium hiranonis* ↓*Bacteroides ovatus*	Neto et al. (51)
	Five-weeks old C57BL/6J male mice (*n* = 9/group)	Mice received either low-fat diet (LFD), high-fat diet (HFD), HFD+ 1% blueberry extract (BL), HFD+ 2% blueberry extract (BH), HFD+ 1% cranberry extract (CL), or HFD+ 2% cranberry extract (CH) for 24 weeks	-Cranberry and blueberry: ↓*Rikenella* and Rikenellaceae. ↑*Lachnoclostridium, Roseburia* and *Clostridium* sp. ↑ SCFA production	Liu et al. (52)
	6-weeks old C57BL/6J male mice (*n* = 12/group)	Mice received either a Chow or a High Fat-High Sucrose (HFHS) diet for 9 weeks to induce obesity Mice fed HFHS diet were then treated either with cranberry polyphenols (200 mg/kg) (HF+CP), Agavins (HF+AG), or the combination of CP and agavins (HF+CP+AG) for 9 weeks	-HF+CP, HF+AG or HF+CP+AG: ↑Bacteroidetes and ↓Actinobacteria phyla ↓ Eggerthellaceae and Ruminococcaceae -HF+CP+AG: ↑*Lactobacillus, Bacteroides acidifaciens, Faecalibaculum rodentium* and *Muribaculum*-HF+CP: ↑*A. muciniphila* (5X)	Medina-Larqué et al. (53)
Clinical	Double-blind, randomized and placebo-controlled trial Healthy women with history of recurrent Urinary Tract Infection (UTI), *n* = 70	240 ml/d of cranberry beverage or placebo for 24 weeks	*↓ Flavonifractor* OTU41 - *E*. *coli* abundance not changed after cranberry consumption	Straub et al. (54)
	Prospective, randomized, double-blind, controlled, 12-week crossover study Obese otherwise healthy men and women with BMI ≥ 30 kg/m^2^ (*n* = 36)	Participant were challenged with 975 mg of aspirin prior to intervention 480 mL of artificially sweetened, low-calorie cranberry beverage or control daily for 2 weeks	↑*Faecalibacterium prausnitzii* and *Eggerthella lenta*	Solch-Ottaiano et al. (55)

## 3. Cranberry PAC, the microbiota, PAC microbial metabolites and the anti-inflammatory effects of cranberries

*In vivo* studies that have demonstrated effects of cranberry in models of inflammation have implicated the microbiota in these effects. For example, in a mouse colitis model, whole cranberry decreased inflammatory cytokines in the colonic mucosa (48, 56) and whole cranberry powder was able to counteract the microbiota alterations by restoring *Lactobacillus* and *Bifidobacterium* with implications for better repair and protection from cancer (48). In mouse models of diet-induced obesity, PAC-rich extracts improved metabolic parameters (reduced liver triglyceride accumulation, improved insulin sensitivity) during high fat-high sucrose feeding (50) and shifted gut microbiota from a *Firmicutes/Ruminococcus* enterotype enriched in opportunistic bacteria to an enterotype enriched in *Lactobacillus* and *Akkermansia*, while boosting butyrate-producing bacteria (57). Interestingly, polyphenol-degrading bacterial families such as *Eggerthellaceae* were also favored, suggesting that polyphenol metabolism accompanies microbiota shifts that are favorable to metabolic health. Causal relationships are difficult to demonstrate. Though, studies that investigate the effects of microbial metabolites of cranberry polyphenols would be helpful. Similarly to its parent compound PAC (58), DHPPA was also reported for its anti-inflammatory properties *in vitro* [reviewed in Mithul Aravind et al. (59)]. Moreover, one *in vivo* study showed that pre-treatment with DHPPA at a dose of 30 mg/kg caused a significant decrease of abdominal contractions induced by acetic acid administration in rats (60). Interestingly, protective effects of PAC microbial metabolites DHPPA and HPPA were also seen against p-cresol, a toxic compound produced by the gut microbiota from l-tyrosin. Both metabolites prevented p-cresol-induced alterations of cell membrane integrity and cell death *in vitro* (61). Though, *in vivo* studies testing the direct effects of PAC metabolites in the context of chronic inflammation are lacking. In terms of the mechanisms that may underlie anti-inflammatory effects of PAC and its metabolites, gene expression regulation has been consistently described. This includes genes regulating the NF-kB pathway and downstream cytokines (58, 59).

## 4. MicroRNAs and intestinal homeostasis

MiRNAs are a class of small (~22 nucleotides in length), non-coding RNAs that are highly conserved across species and regulate gene expression at the post-transcriptional level (62). Thus far, 2,693 miRNAs have been annotated in humans (miRBase release 22.1) (63) and it is estimated that over 60% of human protein-coding genes are targeted by miRNAs (64). Overall, the potential impact of miRNAs is markedly pronounced because one miRNA can have hundreds of targets, while multiple miRNAs can also share the same target. In the intestine, lack of mature miRNA forms results in impaired barrier function and an inflammatory phenotype (65, 66). More recently, evidence has emerged supporting a role for intestinal miRNA in host-microbiota crosstalk. We and others demonstrated that miRNA expression is microbiota-dependent in various intestinal regions (4, 6, 67). Intestinal miRNAs can also be found in the lumen (68, 69), where they may regulate bacterial growth and gene expression (69, 70). Luminal miRNAs can be ultimately recovered in the feces (fecal miRNAs). Intestinal miRNA signatures are increasingly being used to discern between states of health and disease. In IBD, miRNA are aberrantly expressed in tissue biopsies, body fluids and feces of patients with Crohn's disease (CD) and active ulcerative colitis (UC) in comparison to healthy controls [reviewed in James et al. (71)], including elevated miR-301a expression acting as a promotor of intestinal and mucosal inflammation (72, 73). Moreover, fecal miRNA signatures correlate with disease activity (74), and recent studies suggest that they may play a role in IBD pathology by shaping the intestinal microbiota [reviewed in Casado-Bedmar and Viennois (75)]. In fact, IBD-associated miR-199a-5p, miR-1226, miR-548ab, and miR-515–5p were found to affect the proliferation of intestinal *E. coli, Fusobacterium nucleatum* and segmented filamentous bacteria (76). MiRNAs also play an extensive role in the progression and proliferation of colorectal cancer, they are associated with resistance to chemoradiotherapy and are involved in the regulation of signaling pathways such as the Wnt/β-catenin and ERK-MAPK pathways [reviewed in Zhang et al. (77)]. MiRNA signatures in tumor tissue, cancer-derived exosomes, stool, and plasma have been explored as predictive indicators to identify the best treatment strategies for patients based on factors including predicted chemoresistance or metastatic properties of the cancer [reviewed in To et al. (78)]. More recently, a panel of circulating miRNAs has been identified as potential biomarkers of gastroenterological cancers [reviewed in Hoshino (79)], celiac disease [reviewed in Felli et al. (80)], and other human infectious diseases [reviewed in Ojha et al. (81)].

### 4.1. Dietary polyphenols and microRNA modulation

Dietary compounds modulate host miRNA expression in various organs including the intestine. The impact of dietary polyphenols on miRNA expression have been studied in different *in vitro* and *in vivo* models (Table 2). Grapeseed PAC, commonly referred to as grape seed proanthocyanidin extract (GSPE), has been studied more extensively compared to PAC derived from cranberries. There is emerging evidence that the biological effects of GSPE depend on miRNA modulation. *In vitro*, GSPE treatment resulted in the differential expression of miR-30b, miR-197, miR-532-3p, and miR-1224-3p in HepG2 hepatoma cells (82). In human glioblastoma stem cells, GSPE in combination with miR-30e transfection led to the induction of apoptosis (83). In lung cancer cell lines and tumor xenografts, GSPE decreased miR-19a/b expression, which inactivated PI3K/AKT signaling and inhibited cell proliferation and induced apoptosis (84). GSPE also inhibited growth of human melanoma cell lines by downregulating the oncomiR miR-106b (85). In THP-1 macrophage cells, GSPE inhibited foam cell formation by decreasing the lipid content in the cells and inducing miR-9, which downregulated the expression of *ACAT1*, encoding for an enzyme that catalyzes intracellular free cholesterol to cholesterol esters (86). In animal models, consumption of GSPE improved plasma and liver lipid profiles, and normalized the overexpression miR-33a and miR-122 in the liver of obese rats (87, 88). Similarly, in C57BL/6 mice fed high-fat diets, GSPE reversed weight gain and dyslipidemia in mice, which was thought to be attributed to the ability of GSPE to downregulate the high-fat diet-induced increase of miR-96 and inhibition of mTOR/FOXO1 signaling (89). Together, these studies suggest that GSPE may interact with specific host miRNAs or modulate their expression to exert a specific biological effect.

**Table 2:** Studies reporting miRNA responses to dietary sources of polyphenols.

**Type of study**	**Experimental model**	**Treatment**	**miRNA response**	**Additional outcomes**	**References**
*In vitro*	HepG2 hepatoma cells	Grape seed proanthocyanidin extract (GSPE), cocoa proanthocyanidin extract (CPE), pure epigallocatechin gallate isolated from green tea (EGCG)	Decreased miR-30b expression (GSPE, CPE, EGCG) Increased miR-197, miR-532-3p and miR-1224-3p expression (GSPE, CPE)	Not assessed	Foo et al. (90)
	Human glioblastoma stem cells and glioblastoma SNB19 cells	GSPE in combination with miR-30e transfection		Inhibition of autophagy and induction of apoptosis	Chakrabarti et al. (83)
	Lung cancer cell lines and tumor xenografts	GSPE	Decreased miR-19a/b expression	Inactivation of PI3K/AKT signaling and inhibited cell proliferation and induction of apoptosis	Mao et al. (84)
	Human melanoma cell lines A375 and Hs294t and tumor xenografts	GSPE	Decreased miR-106b expression (oncomiR)	Inhibition of growth	Prasad and Katiyar (85)
	THP-1 macrophage cells	GSPE	Increased miR-9	Inhibition of foam cell formation by decreasing the lipid content in the cells	Shao et al. (86)
	CCD18Co cells (non-malignant colonic myofibroblasts) challenged with LPS	Cowpea (*Vigna unguiculata*) seeds extracts	Increased miR-126 expression	Downregulation of pro-inflammatory cytokine VCAM-1	Ojwang et al. (91)
	Intestinal myofibroblast CCD-18Co cells	Yaupon holly (*Ilex vomitoria*, Aquifoliaceae) leaves flavonol-rich extract	Increased miR-146a expression	Protection from inflammation	Noratto et al. (92)
	Human esophageal adencarcinoma cell lines JHAD1, OE19, OE33	Cranberry PACs	Increased miR-410 and miR-520d-5p; decreased miR-202, miR-516a-3p and miR-586	Affected pathways related to cancer, DNA damage related pathways, and immune response pathways (*in silico* predictions)	Kresty et al. (5)
	Caco-2BBe1 intestinal cells challenged with IL-1β	Cranberry PAC-rich extract and HPPA and DHPAA metabolites	Several up or down regulated miRNAs; mitigation of inflammatory miRNA responses	Between both metabolites, top affected pathways included EGFR1, epithelial-to-mesenchymal transition regulators, PI-3K/AKT/mTOR signaling, and microRNAs in cancer (*in silico* predictions)	Lofft et al. (93)
*In vivo*	Six-weeks old obese Wistar female rats (*n* = 6/group)	GSPE	Normalized the overexpression of miR-33a and miR-122 (liver)	Improved plasma and liver lipid profiles	Baselga-Escudero et al. (88)
	Six-weeks old Wistar male rats (*n* = 3/group)	GSPE	Reduced miR-33a and miR-122 expression in a dose-dependent manner (liver)	Improved postprandial hyperlipemia	Baselga-Escudero et al. (87)
	Three to nine-months old C57BL/6 male mice fed high-fat diet (*n* = 25/group)	GSPE	Downregulation of the high-fat diet-induced miR-96 (liver)	Inhibition of mTOR/FOXO1 signaling. Reversed weight gain and dyslipidemia.	Shi et al. (89)
	Three-weeks old Sprague-Dawley male rats treated with azoxymethane (to induce aberrant crypt foci) (*n* = 10/group)	Plum (*Prunus salicina* L.) beverage (1,346 mg gallic acid equivalents/L)	Increased miR-143 expression (colon)	Reduced number of dysplastic aberrant crypt foci. Downregulated proinflammatory enzymes and AKT, mTOR and hypoxia-inducible factor-1α	Banerjee et al. (94)
	Ten-weeks old Sprague-Dawley male rats treated with DSS (to induce colitis) (*n* = 10/group) CCD-18Co colon-myofibroblastic cells	Pomegranate (*Punica granatum* L.) beverage	Induced miR-145 expression (tumor suppressor) in a dose-dependent manner (colon)	Attenuated colitis via the miR-145/p70S6K/HIF1α axis	Kim et al. (95)
	Ten-weeks old Sprague-Dawley male rats treated with DSS (to induce colitis) (*n* = 10/group) CCD-18Co cells	Mango (*Mangifera Indica* L.; var. Keitt) extract (10 mg GAE/L)	Increased miR-126 expression in a dose-dependent manner	Protection from inflammation	Kim et al. (96)
*In vivo* and clinical	Two-months old C57BL/6 male mice (*n* = 5/group) Mouse small intestinal organoids Randomized, cross-over, placebo-controlled, and double-blind intervention study 20–40 years old subjects receiving 25 mg/d HT as an olive oil extract for 1 week (*n* = 21)	Hydroxytyrosol (HT) supplement	Modulation of several miRNA expression in small intestine and other tissues Increased miR-193a-5p expression in organoids and human PBMC	Increased triglycerides in mice	Tomé-Carneiro et al. (97)

Kresty and colleagues were the first to evaluate the ability of cranberry PACs on miRNA expression profiles in esophageal adencarcinoma and its precursor, Barrett's esophagus tissue as well as EAC cell lines (5). Across the samples tested, five miRNAs (miR-410, miR-520d-5p, miR-202, miR-516a-3p and miR-586) were commonly aberrantly expressed, which were implicated in multiple biological processes, including pathways in cancer, DNA damage related pathways, and immune response pathways (5). Remarkably, PACs inversely modulated 10 miRNAs (let-7b, miR-106b, miR-143, miR-199a, miR-215, miR-223, miR-23b, miR-32, miR-543, and miR-7) dysregulated in esophageal adenocarcinoma or Barrett's esophagus tissues, showing that cranberry PAC may in part normalize the expression of miRNAs in esophageal cancer. However, the extent to which cranberry PAC mitigate the aberrant expression of miRNAs in gastrointestinal cancers and other intestinal inflammatory pathologies is not clear. We recently investigated intestinal miRNA signatures in Caco-2BBe1 intestinal cells -a homogeneous clone of Caco-2 cells with a morphology, stability and brush-border skeleton comparable to the human enterocytes (98)- pre-treated with PACs derived from cranberry, metabolites DHPAA, or HPPA and stimulated or not with IL-1β (93). At homeostasis, each treatment was associated with a distinctive miRNA signature, but the two metabolites exerted a partially shared miRNA response. More miRNAs were altered by the metabolites compared to PAC, proposing the notion that the microbial catabolism of PAC initiated by the gut microbiota could facilitate its miRNA-regulatory effects. Thus, the gut microbiota may affect intestinal miRNAs via its metabolites, and this closely echoes previous findings for microbiota-produced SCFAs (99, 100). The gene targets of miRNAs responding to the metabolites were significantly enriched in many pathways relating to cell growth and development and pathways in cancer (93). PAC-responsive miRNAs, miR-1260b and miR-542-5p, were previously found to be modulated in response to genistein (101, 102) and glyceollins (103), respectively. Metabolites-associated and shared miRNAs were miR-1260a, miR-130a-3p, miR-625-5p, miR-6721-5p and miR-20a. The latter was recently reported to be downregulated in response to resveratrol (104).

Interestingly, PAC and DHPAA reversed the expression of 80% and 15% of miRNAs deregulated by IL-1β, respectively (93), these miRNAs may be key mediators of the anti-inflammatory effects associated with cranberry polyphenols and microbial metabolites (105, 106).

A limited number of studies have investigated the effects of polyphenol metabolites on different biological outcomes and the role of miRNAs as putative mediators. In a study comparing the anti-inflammatory effects of quercetin with its two metabolites isorhamnetin and quercetin-3-glucuronide *in vitro*, pre-incubation of murine macrophage cells with quercetin or its metabolites quercetin-3-glucuronide and isorhamnetin, prior to LPS stimulation decreased mRNA and protein levels of TNF-α, decreased *Il1b, Il6* gene expression, and macrophage inflammatory protein-1α, and thereby NF-κB activation in comparison to LPS stimulation alone. Interestingly, quercetin and its metabolite isorhamnetin reversed the LPS-induced upregulation of miR-155, a miRNA identified as a modulator of inflammatory responses (107). When analyzing the effect of *trans*-resveratrol and its metabolites, *trans*-resveratrol-3-*O*-sulfate, *trans*-resveratrol-3′-*O*-glucuronide, and *trans*-resveratrol-4′-*O*-glucuronide on the expression of adipogenic transcription factors and miRNAs in 3T3-L1 murine adipocytes, Eseberri et al. (108) found that of the nine miRNAs analyzed, only miR-155 responded to different treatments. *Trans*-resveratrol and its metabolites *trans*-resveratrol-3′-*O*-glucuronide, and *trans*-resveratrol-4′-*O*-glucuronide significantly upregulated the expression of miR-155, which led to a decrease of CCAAT enhancer-binding protein-ß gene expression (108). In both studies, the authors mentioned the hepatic and intestinal metabolism of quercetin or trans-resveratrol in general however there was no mention of the role of the gut microbiota. The microbiota and miRNAs may interact to control host gene expression [reviewed in Malmuthuge and Guan (109)], however, the interplay existing between gut microbiota and miRNAs in the presence of dietary polyphenols is still not completely elucidated.

### 4.2. Crosstalk between polyphenols, miRNAs and microbiota: a clinical perspective

**Figure 3:**
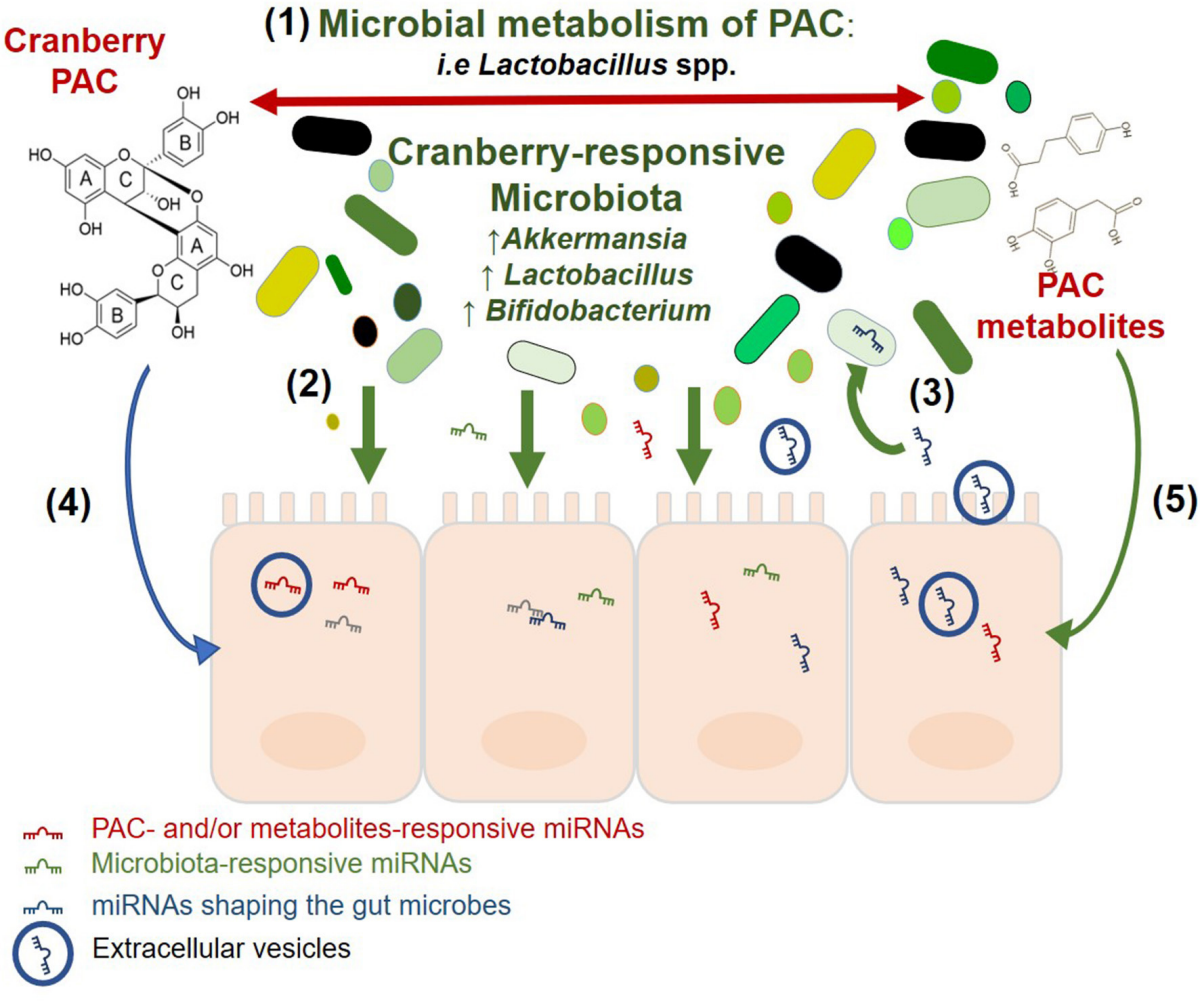
Potential association between cranberry PAC, gut microbiota, and miRNAs. Visualization of the relationships between PAC, microbiota and miRNA in the intestinal ecosystem, based on the available literature. The two-way interaction between cranberry and the gut microbiota as described in the text and reviewed by Prasain and Barnes (110). Gut Microbiota affect the host intestinal response and miRNA signature as previously reported by Dalmasso et al. (4), Singh et al. (6), Peck et al. (67). Intestinal miRNAs may enter resident bacteria, regulate their growth, and shape the gut microbiota [Liu et al. (69), Liu et al. (70)], and Cranberry PAC and their microbial metabolites alter the expression of intestinal miRNAs Lofft et al. (93).

An emerging body of evidence suggests the presence of a tripartite association between cranberry polyphenols, gut microbiota, and miRNAs (Figure 3). These interactions are likely to at least partially underlie the beneficial health effects of cranberries.

#### 4.2.1. Cranberry and gut microbiota

The relationship between cranberry polyphenols and gut microorganisms relies on the microbial metabolism of polyphenols into smaller molecules and bioactive components that can either be involved in trophic interactions or further metabolized by other microbial taxa that possess PAZymes. This cascade of microbial activities modifies polyphenol bioavailability and, as a consequence, the intestinal niche. Cranberry extracts and polyphenols were previously reported to significantly modify the abundance of beneficial bacteria such as *Lactobacillus* and *Bifidobacterium* as well as *A. muciniphila* (48–50, 52, 111). *A. muciniphila* has been suggested as a next-generation probiotic in the context of obesity (112) and diabetes (113, 114). This is aligned with the understanding that cranberry may also exert prebiotic effects, by conferring health beneficial effects to the host via the modulation of gut microbiota. Microbial taxa and their metabolites trigger host responses, including via gene expression regulation. These effects may be post-transcriptional and include miRNAs. *In vitro*, intestinal miRNAs are modulated by cranberry polyphenols and their microbial metabolites (93); intestinal miRNAs also respond to bacterial taxa such as *Lactobacillus* (115, 116) and *Bifidobacterium* (117), which were identified as cranberry-responsive bacteria (48, 118). In tandem, while the gut microbiota affects the expression of miRNA, luminal miRNAs have been shown to affect members of the microbiota (69). Interestingly, the mucin-degrading and cranberry-responsive *A. muciniphila* was found to be targeted by miR-30d, resulting in increased abundance of the bacterium (70). MiR-30d is a member of the miR-30 family which also includes miR-30b and miR-30e, previously reported to be significantly differently expressed in response to GSPE treatment (82, 83). Cranberry polyphenols and their microbial metabolites may mediate prebiotic effects by modulating intestinal miRNAs.

#### 4.2.2. Cranberry and host responses

On the host side, along with microbiota-dependent modulation of the intestinal response, the polyphenolic compounds interact with cell membranes, changing their structure and function. For decades, polyphenols have been considered simple antioxidant molecules capable of interacting with host cells. However, this fact has been questioned (119) and different forms of interactions between host and polyphenols have been suggested (120). It is recognized that the function of polyphenolic compounds is attributed to their ability to interact with membrane surfaces through hydrophobic, electrostatic, or covalent binding [reviewed in Hendrich (121), Reis and De Freitas (122)]. These interactions with cell membranes may be the basis by which polyphenolic compounds exert their beneficial effects on the host (123–125)]. They may also depend on the nature and function of the proteins involved, enzymatic activities, binding sites, and the interaction between specific sites in DNA and transcription factors. Previous studies reported the impact of polyphenols on molecular signal transduction pathways such as cell migration/proliferation, metabolic disorders, oxidative stress as well as inflammation cascades (126). As described above, the beneficial effects of polyphenols also involve the regulation of cell signaling (127, 128), since they act as regulation factors at the transcriptional (gene expression) (58, 129), and posttranscriptional (microRNAs) levels that affect different processes such as cell growth and apoptosis (130). Many *in vitro* and *in vivo* studies support the capacity of polyphenols to modulate cell functionality through the modification of gene expression and protein levels (58, 129, 131) or by an epigenetic mechanism involving miRNAs (5). An emerging body of evidence indicates that miRNAs serve as mediators in regulating polyphenols′ beneficial effects [reviewed in Milenkovic (132), Corrêa et al. (133), Majidinia et al. (134)] and targeting miRNAs could be a novel strategy for inflammation and disease.

#### 4.2.3. Clinical perspective

In clinical practice, the number of intervention studies of cranberry has increased in recent years. Many of these studies focused on the beneficial effects of cranberries as a whole food primarily on outcomes related to UTIs and *H. pylori* infections. Only few studies have explored the microbial response to cranberry intervention. The studies are inconsistent in terms of populations studied and the cranberry products administered, which were mainly cranberry juice or powders, the equivalent dose of polyphenols and the duration of intervention and detection, as well as biomarkers used, either for health outcomes or for microbial metabolism of the cranberry polyphenols. To the best of our knowledge, no study has explored the miRNA-mediated effect of cranberries in a clinical setting. The use of miRNAs may open new avenues in clinical studies. In particular, the study of miRNA signatures in response to cranberry and/or its components in various human samples, such as plasma, urine or feces, in health and disease, may help identifying new biomarkers of administration and response. For example, Seo and colleagues analyzed miRNA signatures in mice exposed to a high-fat and high-fructose diet (HFrD) containing or not 5% polyphenol-rich wine grape seed flour (GSF) (135). MiR-129-5p was identified as significantly affected by GSF in both blood and feces and strongly correlated with biomarkers of obesity such as body weight gain, liver weight, adipose tissue weight, triglyceride, total cholesterol, and HDL (135). The study identified for the first time a potential biomarker for monitoring grape seed flour efficacy.

With regard to cranberry, some miRNAs that were found to respond to PACs, have previously been proposed as fecal or serum biomarkers of gastrointestinal diseases. For example, miR-1260b and miR-542-5p were previously found to be upregulated in CRC and other cancers and suggested to be used as predictive markers (136–139). Interestingly, these miRNAs were found to be downregulated in response to PAC (93). Of the cranberry metabolites-responsive miRNAs in the intestine, miR-130a-3p, found to be upregulated in response to HPPA and DHPAA, is also identified as an anti-tumor miRNA (140). In a similar pattern, the HPPA- and DHPAA-induced miRNA, miR-625-5p, is known to serve as a tumor suppressor and its overexpression has been shown to inhibit the proliferation of gastric cancers (141). These findings suggest that selected miRNAs could be used as potential biomarkers to monitor cranberry intervention efficacy or as a target to mitigate or prevent disease.

## Author contributions

AT, ZL, and EC conceived the review and drafted the manuscript. AT, ZL and BL-I conducted the literature search and constructed the tables. AT and BL-I created the figures. EC supervised this work. All authors edited, revised, read, and approved the final manuscript.
